# The effect of family policies and public health initiatives on breastfeeding initiation among 18 high-income countries: a qualitative comparative analysis research design

**DOI:** 10.1186/s13006-017-0122-0

**Published:** 2017-07-27

**Authors:** Amanda Marie Lubold

**Affiliations:** Department of Multidisciplinary Studies, Indiana State University, Holmstedt Hall, 290, Terre Haute, IN 47809 USA

**Keywords:** Breastfeeding initiation, Comparative analysis, Public health initiatives, Family policies

## Abstract

**Background:**

The objective of this study is to examine the effects of macro-level factors – welfare state policies and public health initiatives – on breastfeeding initiation among eighteen high-income countries.

**Methods:**

This study utilizes fuzzy-set Qualitative Comparative Analysis methods to examine the combinations of conditions leading to both high and low national breastfeeding initiation rates among eighteen high-income countries.

**Results:**

The most common pathway leading to high breastfeeding initiation is the combination of conditions including a high percentage of women in parliament, a low national cesarean section rate, and either low family spending, high rates of maternity leave, or high rates of women working part-time. The most common pathway leading to low breastfeeding initiation includes the necessary condition of low national adherence to the Baby-Friendly Hospital Initiative.

**Conclusion:**

This research suggests that there is a connection between broad level welfare state polices, public health initiatives, and breastfeeding initiation. Compliance with the WHO/UNICEF initiatives depends on welfare regime policies and overall support for women in both productive and reproductive labor.

## Background

Breastfeeding is the optimal source of infant nutrition and has been linked to positive health outcomes in early childhood and into adulthood [1–3]. In Western, high-income Organisation for Economic Cooperation and Development (OECD) countries, however, breastfeeding initiation ranges from 44% to 99% [4] This wide range raises questions about what enables or constrains breastfeeding in different national settings. The purpose of this paper is to examine the case of both public health efforts and welfare state policies as explanations of the breastfeeding variation among eighteen high-income, OECD countries.

Sociologists and economists have only recently begun to study breastfeeding as an outcome of social and political processes in both welfare state and public health research. Galtry [5] examined the effects of labor market policy and socio-cultural factors on breastfeeding rates in three high-income, OECD countries: Sweden, the United States, and Ireland. Cattaneo et al., [6] examined the current situation of breastfeeding in 29 European Union member-states and affiliates, in advance of the European Union (EU)-funded “Promotion of Breastfeeding in Europe” project. The latter authors found that labor market policies are a key predictor of breastfeeding rates in the EU countries. Cattaneo et al. [6] also noted that “different social and cultural determinants, as well as flawed policies and unequal support among and within health-care systems, could also explain differences in breast-feeding rates. But it is definitely difficult to understand why initiation and duration of breast-feeding vary so much, and more comparative research is needed” [6 (pg 43)]. Following that recommendation from Cattaneo et al., the research presented in this paper contributes to the small but growing body of comparative research on breastfeeding, labor market policies, and public health policies by analyzing breastfeeding outcomes in 18 high-income OECD countries.

The World Health Organization (WHO) Global Strategy for Infant and Young Child Feeding examines the four WHO/United Nations Children’s Fund (UNICEF) recommendations for protection and promotion of breastfeeding worldwide [7]. These recommendations follow that of the Innocenti Declaration, a 1990 WHO/UNICEF declaration that stated all member countries should adhere to four policies, including developing national breastfeeding policies and coordinators, ensure that all hospitals follow the Ten Steps to Healthy Breastfeeding, ensure all member-states follow the International Code of Marketing of Breast-Milk Substitutes, and ensure all member-states enact legislation to protect the rights of working mothers [7].

Yngve and Sjostrom [8] performed a comprehensive study on the degree to which EU member-states complied with the WHO/UNICEF recommendations. They found vastly differing breastfeeding rates, along with varying degrees of compliance with the Innocenti Declaration. For example, they found that few countries have both a national breastfeeding coordinator and a national plan of action on breastfeeding, and that there is a great deal of variation in both initiation and duration of breastfeeding, even within similarly-situated countries. In addition, they found that breastfeeding rates are difficult to compare country to country because of varying definitions and operationalization of breastfeeding, including what is considered “exclusive” breastfeeding [8]. Finally, they found that there is very little consistency in reporting of breastfeeding statistics in many countries because, unlike demographic characteristics like fertility, breastfeeding rates are not regularly reported and catalogued [8].

Cattaneo et al. [6] built upon Yngve and Sjostrom’s study, finding much of the same variation. In 2003, they distributed a questionnaire to key personnel in 18 countries: the 15 EU then-member states and Norway, Iceland, and Sweden. The questionnaire asked different actors – governmental agencies, public health institutions, and NGOs – to report the state of breastfeeding in their countries [6]. The survey asked about adherence to the Baby-Friendly Hospital Initiative, adherence to the International Code of Marketing of Breastmilk Substitutes, the degree to which volunteer groups are active in breastfeeding support, and the rates of both exclusive and complementary breastfeeding. Again, the survey found wide variation in adherence to public health initiatives across countries, as well as a great degree of variation in breastfeeding initiation and duration [6].

### Theoretical contributions

One of the theories this paper examines with respect to breastfeeding variation is family policies within broader welfare state theories. Family policies, broadly defined, refer to any regulation of social and economic life relating to both families and/or the interaction between families and other social institutions [9, 10]. Examples of family policies include subsidized childcare, public spending on family benefits, and paid parental leave. Esping-Andersen [11] nested family policies within a broader welfare state classifications system based on differing arrangements of markets, the state, and families. Esping-Andersen [11] identified three welfare state regimes: social-democratic, conservative, and liberal. The social-democratic welfare regime, present in most Scandinavian countries, offers a system in which vast social protections are extended to working-class and middle-class families, and the state provides a variety of family supports to all citizens [11]. Conservative welfare states, including Germany, Austria, and Italy, provide some social supports and financial benefits to mothers, but fewer universal benefits to all citizens. Thus, they offer benefits that exclude non-mothers. Also, the level of support for family work in the form of subsidized or public day care is very limited [11]. Countries in the conservative regime offer long leaves that could encourage motherhood, but then effectively keep mothers out of the labor market because of their low level of support for child care. As a result, they present reproductive labor and productive labor as an either/or choice for women. The liberal regime includes the United States, the United Kingdom, and Canada. In the liberal regime, the state provides very few social supports and financial benefits to families, instead leaving those supports and benefits up to the markets [11]. The few state benefits that are available are typically means-tested and restricted to individuals with great need. Thus, the liberal regime does not support reproductive labor but rather takes a laissez-faire approach.

Welfare state policies provide a useful lens through which to examine breastfeeding outcomes. However, they are not the only predictive factor. Public health policies, including the application of international-level initiatives, have an important role in breastfeeding outcomes [12, 13]. More specifically, the ways in which individual countries have adopted policy recommendations from the World Health Organization (WHO) and the United Nations Children’s Fund (UNICEF) affect the public health climate which either supports or discourages breastfeeding. These policy recommendations, however, do not operate independently from overall welfare state regimes or family policies. In fact, the degree to which welfare states acknowledge the value of care as a social good, and support that care, directly influences the degree to which externally-developed public health recommendations are implemented.

The Baby-Friendly Hospital Initiative (BFHI) is a component of the four-part Innocenti Declaration. Myriad research has shown that the BFHI has a significant, positive effect on breastfeeding initiation. For example, Kramer et al. [12] conducted a landmark Promotion of Breastfeeding Intervention Trial (PROBIT) in the republic of Belarus. They found that in a randomized trial of BFHI interventions in maternity hospitals in Belarus, infants who received the interventions were more likely to breastfeed at 12 months than infants in non-BFHI facilities [12].

Similarly, Merten, Dratva, and Ackermann-Lebrich [13] studied breastfeeding duration among mothers who gave birth in both Baby-Friendly hospitals and non-Baby-Friendly hospitals in Switzerland in 1994 and 2003. They found that breastfeeding duration had been increasing overall, but women who gave birth at Baby-Friendly hospitals experienced greater gains in breastfeeding duration than those who did not. In Norway, many hospitals were transitioning to the WHO’s recommended “Ten Steps to Health Breastfeeding” even before the BFHI was developed. Endresen and Helsing [14] found that Norway’s hospitals and maternity wards were overwhelmingly following the Ten Steps as early as 1991. In fact, breastfeeding at 12 weeks in Norway was greater than 80% in 1991, compared to only 30% in 1968 [14].

It begs the question, then, how an intervention like the BFHI can be combined with other policy initiatives to increase rates of breastfeeding. Lutter and Morrow [15] examined the degree to which the implementation of the WHO/UNICEF Global Strategy for Infant and Young Child Feeding affects exclusive breastfeeding rates over a 10 to 20 year period in various countries. The Global Strategy includes nine operational targets relating to worldwide breastfeeding, and includes the reaffirmation of goals of the Innocenti Declaration [15]. The WHO developed the World Breastfeeding Trends Initiative (WBTi) as a tool to assess national practices and policies [15]. Lutter and Morrow found that countries who implement and adhere to the WHO Global Strategy have shown improvements in exclusive breastfeeding [15]. Perez-Escamilla and Moran tackle the challenge of scaling up effective breastfeeding interventions to a national level. They rely heavily on the theory of Complex Adaptive Systems (CAS) approach of recognizing the interconnectedness of the variety of agents and resources needed to successfully implement health programs at a national level [16, 17]. As breastfeeding policy is well-suited for this CAS lens, it is valuable to understand the intricacies *and* connections between the WHO/UNICEF Global Strategy and the broader welfare state structures those policies must operate in. As such, the analysis in this paper combines an understanding of both implementation of pieces of the WHO/UNICEF Global Strategy and the welfare state policies and characteristics of high-income countries. The specific WHO/UNICEF policies this analysis examines are the participation in the BFHI and maternity protections for women, measured as weeks of paid maternity leave guaranteed to women. These two indicators are used because of the availability and comparability of the measures over the country selection.

To examine the effects of both public health initiatives and welfare state policies on breastfeeding initiation among eighteen high-income countries, fuzzy-set Qualitative Comparative Analysis methods was used. The goal of the current research was to examine the combinations of conditions leading to both high and low national breastfeeding initiation rates.

## Methods

### Sample selection

Table 1 displays the countries used in the analysis as well as the breastfeeding initiation rates, on or about 2005. The country selection is based on prior research and the availability of comparable breastfeeding data. Note that data were not available for each country for the same year. Data availability on breastfeeding, in fact, is a common problem throughout comparative breastfeeding literature. In keeping with previous research, this paper includes breastfeeding initiation information on or around 2005. Table 1 displays the exact year and source. In keeping with similar studies of breastfeeding, child health outcomes, and maternity leave [18, 19] only high-income, OECD countries were included in order to minimize variation in other influences on breastfeeding rates, including cost of commercial formula, availability of clean water, and access to commercial formula. The country selection was also driven by research by Catteneo et al. [6, 20] who were among the first to perform a comprehensive study of breastfeeding outcomes and adherence to WHO initiatives to increase breastfeeding rate.

**Table 1:** Breastfeeding initiation, year and source

Country	Percent Initiation	Year	Source
Australia	92	2004	[4]
Austria	93.2	2005	[41]
Belgium	65.9	2007	[4]
Canada	84.5	2003	[4]
Denmark	98	2001	[4]
Finland	93	2005	[4]
France	63	2003	[42]
Ireland	47.7	2005	[43]
Italy	81.1	2005	[4]
Japan	96.6	2005	[4]
Netherlands	79	2005	[4]
New Zealand	87.8	2006	[4]
Norway	99	2006	[4]
Portugal	91	2003	[4]
Spain	77.2	2006	[4]
Sweden	97.6	2006	[4]
United Kingdom	77	2005	[4]
United States	74.2	2005	[4]

The eighteen countries included in the analysis represent a wide range of geography, economy, and culture, but are all members of the Organization for Economic Co-operation and Development (OECD), a global organization made up of 34 member-states dedicated to “promote policies that will improve the economic and social well-being of people around the world” [21].

### Outcome variable

*Breastfeeding initiation* refers to the percentage of women who ever breastfed, even just once. Most countries collect data on breastfeeding initiation, even if they use different data collection procedures. The OECD complies these data in their cross-national Family Database [4]. Initiation ranges from 99% to 47.7% among the countries in this analysis.

### Explanatory variables

*Percentage of Baby-Friendly hospitals* is operationalized as the percentage of hospitals with maternity wards that are certified as meeting the WHO/UNICEF criteria for baby-friendly, meaning they follow the Ten Steps to Healthy Breastfeeding.

*Weeks full-time equivalent paid maternity leave* addresses the WHO/UNICEF recommendations for maternity protections for women [7]. Maternity leave policies vary in both scope and breadth across the countries in the analysis. Broadly speaking, many countries have “parental leave” policies that can be either maternal leave, specifically to be taken by the mother, paternal leave, specifically to be taken by the father, or parental leave, which can be taken by either the mother or the father [22]. Maternity leave generosity is measured in two ways: the amount of time mothers can take from work without fear of losing their job, and the amount of compensation that women receive as a portion of their salary while they are taking leave. Most countries offer both: job-protected leave that is paid at a portion of the salary. One notable exception is the United States, which provides job-protected leave only [23]. Maternity leave is an important predictor of breastfeeding outcomes cross-nationally because mothers in countries with generous maternity leave policies may have more opportunity to spend time at home with their infants, thus reducing the opportunity costs and any financial penalty associated with breastfeeding [5, 18, 24, 25]. This analysis follow Ray [22] and Ray, Gornick, and Schmitt [23] and operationalize maternity leave as the number of weeks of full-time equivalent paid leave. The number of weeks of full-time equivalent (FTE) leave is calculated by multiplying the wage replacement rate by the number of weeks of job-protected leave. For example, if a country provides women with 50 weeks of leave, paid at 50% of her salary, the number of weeks FTE maternity leave would be 25.

*Female part-time employment rate* is calculated as the percentage of employed women between the ages of 15 and 64 who are working part-time. This analysis uses the International Labour Organization definition of less than 30 h per week [26]. Examining the female part-time employment rate will help to operationalize a key part of the theory – that women in the one and a half breadwinner welfare regime are pinched at both ends.

*Cesarean section rate* is operationalized as the percentage of women delivering via Cesarean section, whether planned or unplanned. Cesarean section rate is rising among high-income countries, and is contraindicated to several of the Ten Steps to Healthy Breastfeeding [7]. For example, women who give birth via Cesarean section often will not have initial skin-to-skin contact with their infant until after the recommended one hour time period [27]. The rise of Cesarean sections and the associated consequences of late breastfeeding initiation require the addition of Cesarean section rate as a predictor variable.*Public Spending on Healthcare*. This variable is operationalized as the “sum of public and private health expenditure. It covers the provision of health services (preventive and curative), family planning activities, nutrition activities, and emergency aid designated for health” [28].

*Public spending on family benefits* is operationalized as a percentage of the country’s overall GDP. As part of a welfare state model, many countries provide families with various subsidized services or cash transfers [4]. Subsidized services include low-cost childcare, provisions for needy families, and early childhood education [4]. Specifically in this analysis, “family benefits” are divided into three categories:

1. Child-related cash transfers to families with children, including cash allowances for having children, public income payments during maternity and paternity leave, and income support for single parent families [4]
2. Public spending on services for families with children, including financing and subsidizing childcare and early childhood education, public spending for residential facilities, and spending on family services such as home care for needy families [4]
3. Tax breaks for families, which include tax exemptions, child tax allowances deducted from gross income, and child tax credits [4]

*Percentage of women in parliament*. is operationalized as simply the percentage of women in either the parliament or the upper house of a bicameral system during the given year. Kittilson [29] and others [30, 31] find that women in governmental positions tend to drive votes on issues such as maternity leave, welfare reform, and other women-specific legislation [32].

Finally, this analysis controls for *fertility rate*, operationalized as “the number of children that would be born to a woman if she were to live to the end of her childbearing years and bear children in accordance with current age-specific fertility rates” [33]. Previous research examining welfare state policies and child health outcomes have controlled for fertility rate [18, 19]. Ruhm [19] has suggested that controlling for fertility rate allows the researcher to look at economies of scale; that is, cost advantages that are provided when more of the product is produced.

### Analysis: Fuzzy-set qualitative comparative analysis (fsQCA)

Qualitative Comparative Analysis (QCA) is a method developed by Ragin that provides an analytic tool to bridge the gap between case-oriented and variable-oriented research [34]. Case-oriented research involves in-depth, small-n studies where many aspects of an individual case are examined and individual cases are compared and contrasted. Variable-oriented research falls on the other end of the spectrum, and is most common in survey-type research, where researchers examine one or two dependent variables and attempt to explain as much variation as possible using large datasets [34]. QCA seeks to “examine similarities and differences across many cases while preserving the integrity of cases as complex configurations [37 (pg 38)]. QCA uses the logic of set relations to address causal complexity and causal configurations in comparative research. Causal complexity is often present in social research – this means that outcomes do not arise from a single source, or cause, but rather by a combination of conditions that operate with each other to produce the outcome. In large, variable-oriented analysis, this type of complexity can be approximated by using interaction terms, but interaction terms fail to capture any nuance. That is, interaction terms assume the interaction is multiplicative, when in reality, two causes just may need to be present together, in any form, to produce the outcome [34, 35].

Necessity and sufficiency are two terms that are important in a QCA analysis. A necessary condition is one in which the causal condition is present in all instances of the outcome. A sufficient condition is one that, when present, always leads to the outcome; that is, it is sufficient on its own to produce the outcome, but doesn’t necessarily have to be present in all instances of the outcome [34].

Once necessity and sufficiency are determined, the QCA researcher can identify the results, keeping in mind simplifying assumptions. Simplifying assumptions take advantage of the fact that there is limited diversity in causal configurations, meaning that some of the logically possible configurations actually don’t exist in the data [34]. The pathways that remain after the simplifying assumptions are taken into account then form the basis of the explanations of causal complexity. These pathways are best explained in set-theoretic logic; for example, high membership in a set of one causal condition combined with low membership in the set of a second causal condition may lead to an outcome.

### Calibration of fuzzy sets

In a fuzzy-set QCA analysis, membership of cases in the fuzzy set specification are calibrated by the researcher. Fuzzy sets represent a fine-grained degree of membership in the set, and fuzzy set scores for each case range from 0 to 1. Calibration is based on theoretical considerations, prior research, and conceptualization of membership in the set. A fuzzy set score of 0.05 is full non-membership in the set, a score of 0.5 is the crossover point, and a score of 0.95 is full membership in the set. It is important to note that fuzzy sets are not simply turning a variable into a continuous variable – they are instead used to determine degree of membership in a set, and the construction of fuzzy sets is based on theory and prior research [34].

Table 2 shows the criteria used for the fsQCA analysis in the current study.

**Table 2:** fsQCA calibration

Variable	Full Membership	Crossover	Full non-membership
Breastfeeding Initiation	95%	75%	50%
Weeks Full-time Equivalent (FTE) Maternity Leave	35	18	3
Percent Baby-Friendly Hospitals	75%	30%	5%
Spending on family benefits - % Gross Domestic Product (GDP)	4%	2.6%	1.5%
Percentage Cesarean Births	32%	22%	14%
Fertility rate, Total	2	1.8	1.4
Percentage of Women in Parliament	40%	29%	11%
Female Part-Time Employment Rate	50%	30%	15%
Health Spending, % GDP	12%	9%	8%

### Recipes

In evaluating QCA solutions, both coverage and consistency are important. Coverage, scored from 0 to 1, refers to how much of the outcome is explained by each solution term and by the entire solution. The total solution coverage is the proportion of the membership in the outcome variable that can be explained by the complete solution (all individual causal “recipes”) [36]. Raw coverage is the proportion of the membership in the outcome that can be explained by each causal recipe in the solution. Raw coverage can include cases that are covered by more than one solution. Unique coverage, on the other hand, is the proportion of cases in the sample that are only covered by the one solution. Of note is the terminology “causal conditions.” In QCA, a “causal condition” is the preferred term over “independent variable” due to the principles of logic and Boolean algebra used in the analysis [37]. Schneider and Wagemann note that “terminological differences can sometimes lead to stylistic problems and substantive confusion,” thus necessitating a note n the manuscript ([37], pg 20).

Consistency in the QCA solutions refers to the degree to which membership in the solution is a subset of membership in the outcome [36]. It also is measured from 0 to 1. A case is considered consistent with each solution term if membership in the solution term is less than or equal to membership in the outcome [36]. For the whole solution consistency, we measure the degree to which membership in the set of solution terms is a subset of membership in the outcome.

The key is to balance consistency and coverage; a solution consistency score of 0.8 is considered meaningful, and a consistency score of 0.9 is highly significant [38]. The QCA algorithm requires the user to specify the criteria used to exclude and code configurations so that logically irrelevant conjunctions are eliminated [39]. In this analysis, 0.8 was used as the threshold for consistent subsets of the outcome.

## Results

Note that because there are no *necessary* conditions for high breastfeeding initiation at the national level, there are several very different pathways that lead to membership in the set of countries with high breastfeeding initiation.

### Outcome variable: Membership in the set of countries with high breastfeeding initiation

**Fig. 1:**
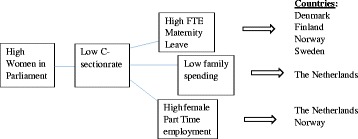
Causal conditions leading to high breastfeeding initiation, solutions 1, 2, and 3

**Fig. 2:**
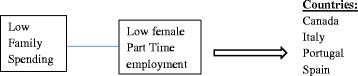
Causal conditions leading to high breastfeeding initiation, solution 4

**Fig. 3:**
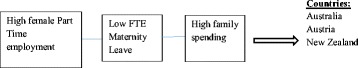
Causal conditions leading to high breastfeeding initiation, solution 5

As Table 3 shows, there are six pathways leading to membership in the set of countries with high breastfeeding initiation. The solutions can be displayed graphically in three models (Figs. 1, 2 and 3):

**Table 3:** fsQCA coverage and consistency

	Raw coverage	Unique coverage	Consistency
1 ~Female part time employment* ~ family spending	0.395	0.200	0.876
2 FTE* ~ c-section* ~ family spending	0.220	0.0282	1.00
3 Women in parliament*FTE* ~ c-section	0.399	0.157	0.983
4 Female part time employment* ~ FTE*family spending	0.307	0.107	0.816
5 Women in parliament* ~ c-section* ~ family spending	0.180	0.000	1.00
6 Female part time employment*women in parliament* ~ c-section	0.264	0.00549	0.838
Solution coverage	0.833		
Solution consistency	0.852		

Outcome variable: high breastfeeding initiation (intermediate solution)

Note 1: The intermediate solution makes only basic, or “easy” assumptions about remainders. It is the mostly commonly used solution in fsQCA [44]

Note 2: the tilde (~) symbol before a variable indicates that there is *low* adherence to that particular variable

Note 3: The asterisk symbol (*) means "the combination of" the associated conditions

**Fig. 4:**
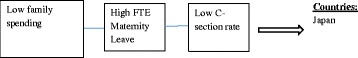
Causal conditions leading to high breastfeeding initiation, solution 6

The first three solutions (Fig. 1), including Denmark, Finland, The Netherlands, Norway, and Sweden all involve countries with both a high level of women in parliament and a low cesarean section rate. The set of Scandinavian countries, Denmark, Finland, Norway, and Sweden, all with very high breastfeeding initiation, also share in common high levels of maternity leave. The fourth solution for high breastfeeding initiation includes Canada, Italy, Portugal, and Spain. These countries share a very different set of characteristics; they have low family spending but a low female part-time employment rate. The fifth solution again clusters different sets of countries. Australia, Austria, and New Zealand all have a high female part-time employment rate, high family spending, but low levels of maternity leave. Finally, Japan has high breastfeeding initiation but fits into a solution by itself, with low family spending, high maternity leave, and a low Cesarean section rate (see Fig. 4 and Table 4 for fuzzy set scores).

**Table 4:** Raw and fuzzy set scores, predictor variables

Country	Weeks FTE Maternity Leave		Female Part-Time employment Rate		Spending on Family Benefits (% GDP)		Percentage Cesarean Births		Percent Baby-Friendly Hospitals		Health Spending, % GDP		Percentage of Women in Parliament		Fertility Rate, Total	
	Raw Value	Fuzzy Set Score	Raw Value	Fuzzy Set Score	Raw Value	Fuzzy Set Score	Raw Value	Fuzzy Set Score	Raw Value	Fuzzy Set Score	Raw Value	Fuzzy Set Score	Raw Value	Fuzzy Set Score	Raw Value	Fuzzy Set Score
Australia	0	0.03	38.7	0.79	2.83	0.62	30.3	0.92	8	0.07	8.5	0.18	27.4	0.43	1.8	0.5
Austria	16	0.4	30.1	0.5	2.95	0.68	27.1	0.82	7	0.06	10.4	0.8	33.9	0.79	1.4	0.05
Belgium	13.9	0.31	33.1	0.61	3.45	0.86	15.9	0.09	0	0.03	10	0.73	34.7	0.83	1.8	0.5
Canada	28.6	0.87	26.9	0.35	1.55	0.05	26.3	0.78	1	0.03	9.8	0.69	21.1	0.21	1.5	0.1
Denmark	18.6	0.53	23.9	0.23	3.9	0.94	21.4	0.44	18	0.19	9.8	0.69	38	0.92	1.8	0.5
Finland	29	0.87	14.8	0.05	3.29	0.81	16.3	0.11	14	0.13	8.4	0.14	37.5	0.91	1.8	0.5
France	19.8	0.58	22.6	0.19	3.98	0.95	18.8	0.23	0	0.03	11	0.88	12.2	0.06	1.9	0.82
Ireland	20.8	0.62	34.6	0.67	4.24	0.97	26.2	0.78	0	0.03	7.6	0.01	13.3	0.07	1.9	0.82
Italy	25.1	0.78	28.8	0.44	1.58	0.06	38.2	0.99	0	0.03	8.7	0.29	11.5	0.05	1.3	0.02
Japan	26	0.8	31.7	0.56	1.48	0.05	17.4	0.15	6	0.05	8.2	0.08	7.1	0.03	1.3	0.02
Netherlands	16	0.4	60.7	0.99	2.48	0.42	13.5	0.04	7	0.06	10.9	0.87	37.6	0.91	1.7	0.32
New Zealand	14	0.31	35.1	0.68	3.56	0.89	20.4	0.35	0	0.03	8.4	0.14	28.3	0.47	2	0.95
Norway	38	0.97	32.9	0.61	3.34	0.83	16.6	0.12	60	0.88	9.5	0.62	38.2	0.92	1.8	0.5
Portugal	17	0.45	14	0.04	1.71	0.08	34	0.97	0	0.03	10.4	0.8	19.1	0.16	1.4	0.05
Spain	16	0.4	21.5	0.15	1.77	0.09	25.9	0.76	0	0.03	8.3	0.11	36	0.87	1.3	0.02
Sweden	40	0.98	19	0.1	3.75	0.92	17.3	0.15	97	0.99	9.1	0.52	45.3	0.99	1.8	0.5
U.K.	12.6	0.25	38.5	0.78	4.22	0.97	22	0.5	14	0.13	8.3	0.11	18.1	0.14	1.8	0.5
U.S.	0	0.03	17.8	0.08	1.22	0.02	30.3	0.92	1	0.03	15.8	1	15	0.09	2.1	0.99

### Outcome variable: Membership in the set of countries with low breastfeeding initiation

**Fig. 5:**
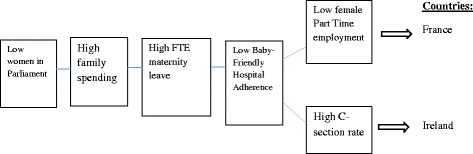
Causal conditions leading to low breastfeeding initiation, solutions 1 and 2

As evidenced in Table 5, there are two pathways in the dataset that leads to low breastfeeding initiation. Figure 5 displays this solution.

**Table 5:** fsQCA coverage and consistency

	Raw coverage	Unique coverage	Consistency
1 ~Female part time employment rate* ~ Women in Parliament*family spending*FTE* ~ BFHI	0.381	0.0819	0.810
2 ~Women in Parliament*C-section rate*family spending*FTE* ~ BFHI	0.366	0.0667	0.803
Solution coverage	0.448		
Solution consistency	0.833		

Outcome variable: low breastfeeding initiation (Intermediate solution)

Note 1: The intermediate solution makes only basic, or “easy” assumptions about remainders. It is the mostly commonly used solution in fsQCA [44]

Note 2: the tilde (~) symbol before a variable indicates that there is *low* adherence to that particular variable

Note 3: The asterisk symbol (*) means "the combination of" the associated conditions

France and Ireland fit into the two solutions for low breastfeeding initiation. In both France and Ireland, there is a low level of women in Parliament, high family spending, high levels of maternity leave, and low adherence to the Baby-Friendly Hospital Initiative. France has a low female part-time employment rate, and Ireland has a high cesarean section rate.

## Discussion

The fsQCA results shed light on the combinations of conditions that lead to both high breastfeeding initiation and low breastfeeding initiation. When looking at high breastfeeding initiation rates, seven countries fit the pathway that includes a high percentage of women in parliament. In the fsQCA analysis, countries in the subset with high female representation in parliament also are in the set of countries with high breastfeeding initiation. The specific variable of women in parliament helps to cluster some of the countries. For example, all of the Scandinavian countries – Denmark, Finland, Norway and Sweden – as well as Austria, the Netherlands, and Spain, have both high female representation in parliament and high breastfeeding initiation rates. In fact, of all the countries with a high percentage of women in parliament, only Belgium does not also have high breastfeeding initiation. This suggests that having high representation of women in the governing body of a country may facilitate female-centered outcomes, notably in this case breastfeeding initiation. This follows existing theory on women in parliament, because countries that are more female-friendly in other ways may have both more female representation in governmental roles and be more accommodating of breastfeeding. In looking at the research question underlying the current study – how do state supports and public health initiatives work together to produce a climate conducive (or not) to breastfeeding? – it makes theoretical sense that having women in parliament will be a significant pathway to high breastfeeding initiation, because carework is more likely to be valued by a government with a higher percentage of women. This raises the possibility that a third factor could be driving the interaction, which is a consideration in future research.

In the set of countries with high breastfeeding initiation, there are several very different pathways. One particularly noteworthy finding is that four of the six pathways include low cesarean section rate. This supports research by Rowe-Murray and Fisher [27] who found that cesarean section birth increases the time between birth and skin-to-skin contact between mother and infant, which is detrimental to early breastfeeding success.

Of particular note in looking at the set of countries with high breastfeeding initiation is that the BFHI was not a significant predictor. However, when looking at the negative outcome – the set of countries with low breastfeeding initiation – the BFHI *was* a significant factor in the solution. For the small number of countries in this study with low breastfeeding initiation rates, having low participation in the BFHI is a necessary condition for having low breastfeeding initiation, meaning that while high participation may not be the driving force for high breastfeeding outcomes, low participation is a negating factor. This suggests that in countries that want to increase breastfeeding initiation, the BHFI may give countries the needed extra “push” to boost breastfeeding initiation to a point where other structural factors can play a larger role. Participation in the BFHI may assume that countries value carework and recognize breastfeeding as a public good.

The fsQCA analysis also allows for an examination at the subset of countries with low breastfeeding initiation because of the asymmetry of set theory. In looking at the set of countries with low breastfeeding initiation, both a policy-level component and a public health-level component were missing. In looking at France and Ireland, they are both part of the conservative welfare regime, which supports the model of the male breadwinner and female caregiver. As such, both countries have high levels of support for female reproductive labor, but do not have a high level of female representation in government, which, it could be argued, demonstrates an overall lack of female-friendliness. This suggests that countries whose polices do not support carework may also not have the ability to develop and implement effective women-centered public health policies. That is, public health policies may require a strong support of women and carework in the welfare state policies before they can operate at the institutional level. The results of the fsQCA analysis also build upon the work of Lutter and Morrow in their analysis of WBTi assessments. This analysis combines some of the WBTi indicators – specifically BFHI, and maternity protection, along with a broader context of policy and the labor market – to further shape the understanding of how policies may be implemented and how that implementation affects breastfeeding initiation [15]. It is difficult to apply policy per se to the outcome; it is more likely to be the successful implementation of this policy.

The use of fsQCA as a method is not without its limitations. Schneider and Wagemann recommend standards for best practices of using QCA analysis, along with the conclusions that can reasonably be drawn from the method [37]. Specifically, they caution that individual pieces of the configurational solution should not be overinterpreted. They also caution that the configurational solution alone is not sufficient for determining causality [37]. In fact, QCA is useful for testing existing theories and exploring new theories [40]. The analysis in this manuscript should be seen as a beginning – a way to connect the influences of public health and policy explanations for breastfeeding outcomes. Ragin [36] cautions that QCA techniques are best for exploring evidence and do not hold the same inferential capabilities as other methods. In the case of France and Ireland, for example, there is only one pathway. This is not causative, per se, but invites further study into the configurations. Indeed, adding additional cases may slightly change some of the pathways, but will add more richness to the analysis.

## Conclusions

This research suggests that there is a connection between broad level welfare state polices, public health initiatives, and breastfeeding initiation. Compliance with the WHO/UNICEF initiatives depends on welfare regime policies and overall support for women in both productive and reproductive labor. For example, Norway and Sweden have high participation in the BFHI and have initiated many parts of the Innocenti Declaration. The Innocenti Declaration, one of the most comprehensive WHO/UNICEF policies targeting breastfeeding, recommends supports for women to engage in reproductive labor, specifically breastfeeding, and includes provisions for women to combine breastfeeding with productive labor in recommending a minimum amount of paid maternity leave. Sweden and Norway also have policies that recognize women as both contributors to the labor market and as valued providers of carework. In these countries, women gain their rights and positions in society through *both* productive and reproductive labor, and carework is supported as a valued public good.

Second, the results of the current study suggest that the absence of BFHI is significantly related to lower breastfeeding initiation. The results of the fsQCA analysis demonstrate that being part of the set of countries with low participation in the BFHI is a necessary condition leading towards being in the set of countries with low breastfeeding initiation.

This study provides a valuable framework from which to understand the relationship between national-level family polices, public health initiatives, and breastfeeding initiation among high-income, Western countries. Future studies may examine breastfeeding duration, changing in breastfeeding rates over time, and the influence of culture and religion on country-level outcomes.

## Abbreviations

BFHI: Baby-Friendly Hospital Initiative
EU: European Union
fsQCA: Fuzzy-set Qualitative Comparative Analysis
FTE: Full-time Equivalent
GDP: Gross Domestic Product
OECD: Organisation for Economic Cooperation and Development
QCA: Qualitative Comparative Analysis
UNICEF: United Nations Children’s Fund
WBTi: World Breastfeeding Trends Initiative indicator
WHO: World Health Organization
